# A multilevel analysis of factors associated with wasting in children one to two years old in Hamadan city

**DOI:** 10.1038/s41598-024-75535-6

**Published:** 2024-11-18

**Authors:** Sara Manoochehri, Zohreh Manoochehri, Fatemeh Torkaman Asadi, Ali Reza Soltanian

**Affiliations:** 1 Department of Biostatistics, Student Research Committee, Hamadan University of Medical Sciences, Hamadan, Iran; 2 Social Development and Health Promotion Research Center, Health Institute, Kermanshah University of Medical Sciences, Kermanshah, Iran; 3 Department of Infectious Disease, School of Medicine, Hamadan University of Medical Sciences, Hamadan, Islamic Republic of Iran; 4 Department of Biostatistics, School of Public Health, Modeling of Noncommunicable Diseases Research Center, Hamadan University of Medical Sciences, Shahid Fahmideh Boulevard, Hamadan, Iran

**Keywords:** Multilevel ordinal models, Malnutrition, Thinness, Failure to Thrive, Pediatrics, Diseases, Health care, Risk factors

## Abstract

The study aimed to identify risk factors for childhood wasting in 1–2 year-olds in Hamadan city, focusing on this age group due to infection and malnutrition risks. Unlike previous cross-sectional studies on children under 5 years old, this longitudinal study tracked weight-to-height changes over time. Data were analyzed from 455 mother-child pairs, aged 1–2 years, collected from health centers and recorded in the Integrated Electronic Health System (SIB). The weight-for-height index, an ordinal response with three categories (wasting, normal, and overweight), was measured several times. A two-level longitudinal ordinal model was used to identify factors associated with wasting. The analysis of data from 230 girls and 225 boys identified several factors associated with wasting: lower birth weight (Adjusted Odds Ratio (AOR) = 0.77), age 12–15 months (AOR = 1.15), lack of health insurance (AOR = 3.09), mother-child residence (AOR = 3.80), maternal height (AOR = 0.92), and age at pregnancy < 24 years (AOR = 4.71). The results of this study showed that most of the factors contributing to childhood wasting can be controlled and prevented. Therefore, implementation of targeted policies and appropriate interventions for mothers before, during, and after pregnancy could reduce the burden of childhood wasting.

## Introduction

Childhood malnutrition is one of the most important public health challenges worldwide, especially in developing countries. Malnourished children are at increased risk of contracting diseases such as measles, diarrhea, and various infectious diseases. Among the various forms of malnutrition, marasmus and kwashiorkor are two forms of severe malnutrition^1^. Three main indicators are used to diagnose malnutrition: wasting, underweight and stunting^2^.

Wasting, an indicator of acute malnutrition, is of particular concern because it carries a high risk of death and is considered a medical emergency^3^. Wasting is defined as weight for height with a z-score less than − 2 standard deviations (-2 SD) from the median of the World Health Organization (WHO) standards for child growth^4^. This condition, which results from inadequate nutrient intake or illness, is associated with loss of fat and muscle tissue and can have long-term consequences for a child’s growth. Wasted children have a weakened immune system, making them more susceptible to infections and various diseases^5^. In addition, this condition can lead to cognitive impairment, reduced learning capacity, poor school performance, and reduced productivity in the future^6^.

The public health burden of wasting is so great that more than 800,000 deaths of children under- five, or 12–13% of all under-five deaths, are attributable to wasting^7^. In 2020, UNICEF reported that 45.4 million children under five suffered from wasting, with 13.6 million whom were severely wasted. This value was 1.5 times higher than the predicted value for 2020. The cause of this increase is likely to be the impact of COVID-19, followed by the unfavorable economic situation of families, disruption of access and affordability of nutrients and essential items^8^. Geographically, Asia bears the highest burden of wasting, home to more than half of the world’s wasting children (69%), followed by Africa with 27%^7^. In Iran, the prevalence of wasting in children under five years of age was reported to be 4.1% in 2018^9^. Specifically, a 2019 study showed that the prevalence of wasting in children aged two to five years in Hamedan was approximately 5%^10^.

The causes of various forms of malnutrition, including child wasting, depend on the complex interactions of various factors such as socio-demographic, environmental, cultural, and political^11^. Identifying risk factors for wasting in children helps physicians and health care workers to identify children with high-risk groups at admission and, in consultation and cooperation with health policy makers, to implement intervention programs and training methods to prevent growth failure and wasting.

In contrast to the studies conducted in this field, which focused on children under 5 years of age, this study examined children between the ages of 1 and 2 years, because there is evidence that the prevalence of wasting in children under 2 years of age is higher due to exposure to infections and malnutrition. For example, the prevalence of wasting in children under 2 years of age is 14%, while this rate is 9% in children between 2 and 4 years of age^12^. On the other hand, most of the studies that have examined the condition of wasting in children have been cross-sectional and do not provide information about the age changes in wasting^13–15^, but in this study, to examine the trend of weight changes over time, the response variable of weight to height was measured several times over time.

## Materials and methods

### Study setting

In this longitudinal study, mothers and children aged 1 to 2 years who visited the health centers of Hamedan city (one of the western cities of Iran) during 2022 to 2023 were examined. The number of health centers in Hamadan city is 24, from which 4 centers were randomly selected for this study. The total number of mothers and children referred to the selected databases was 455 mother-child pairs. A total of 1097 repetitions were studied, including measurements at 12, 18, 15 and 24 months of age. These measurements are proportional to the age of the child, e.g., for a 20-month-old child, 3 measurements are recorded (12, 15 and 18 months). Note that the minimum number of measurements is 2 and the maximum is 4. The Integrated Health System (SIB) was used to collect the necessary information on the study samples. In the SIB system, to provide integrated health services to Iranians, the health information of all people from birth to death is continuously and electronically recorded over time^16^.

### Data collection

The outcome variable investigated in this study is wasting. According to the proposal of the World Health Organization (WHO), the anthropometric index used in the study of wasting is the Weight for Height ***Z*score** (WHZ)^2^, which is classified in Table 1^4^.

**Table 1:** World Health Organization classification for different groups of weight for height *Z* score.

weight-for-height z score	Category of weight-for-height
WHZ > 2 SD and ≤ 3 SD	Overweight
≤ WHZ ≤ + 1z-score − 2z-score	Normal
WHZ ≤–2 SD and ≥–3	Wasting

*WHZ* weight-for-height z score

*SD* standard deviation

The predictor variables related to the child are: sex, birth weight (gr), age (months), type of feeding (breast milk/ formula milk), and preterm birth: A baby born before the thirty-seventh week^17^ (yes/no), birth rank of the child (rank one / two and above), having two or more twins (yes/no), distance from the birth of the previous child (only child, less than two years, more than two years), growth monitoring age (12–15 months, 15–18 months, 18–24 months), health insurance status (yes/no).

The predictor variables related to maternal characteristics are: Maternal age (years), age at pregnancy (years), pre-pregnancy weight (kg), height (cm), pre-pregnancy Body Mass Index(BMI) (kg/m2), level of education (less than diploma/diploma/academic), employment status (unemployed/employed), planned pregnancy (wanted/unwanted), history of abortion (yes/no), type of delivery (caesarean /natural), maternal health status (sick, healthy), place of residence (urban/suburban: tissues that have mainly rural migrants and urban slums^18^).

Children aged 1–2 years from the city of Hamadan who had complete medical records were included in the study. In this study, children who had a genetic disease or congenital anomaly, or whose medical record information was incomplete, were excluded.

## Statistical methods

### Multilevel ordinal logistic regression model

In the present study, to model the factors related to children’s wasting, a two-level ordinal model was used, in which the repeated measures included the weight of children 1 to 2 years old measured during 12 to 24 months (level 1) within children (level 2) of the nest. The two-level ordinal logistic model with random intercept is shown as follows:

1 $$\:logit\left[P\left({Y}_{i}\le\:j\right)\right]={\alpha\:}_{j}+{X}^{{\prime\:}}\beta\:+{u}_{i}\:\text{j}=\text{1,2},.,\text{J};\:{\alpha\:}_{J}=0$$

In Eq. (1), $\:{{\upalpha\:}}_{\text{j}}$ represents the J-1 random intercept for J of the ordinal model. X is the fixed-effects design matrix, β represents the effect of the investigated variables or the vector of regression coefficients, and $\:{u}_{i}$ is the random intercept for the ith child, which has a normal distribution with zero mean. In the present study, $\:{Y}_{i}\:$represents weight groups for height, which are considered waste, normal, and overweight (J = 3)^19^.

To obtain descriptive statistics related to the variables, spss software was used, and to perform calculations related to the generalized linear mixed model, R software version 4.0.3 package clmm2 was used. In this study, the significance level of the tests was considered to be less than 0.05.

#### Ethical approval and consent to participate

This is an observational study in which the data were extracted from the SIB system through file review. Since the data were obtained from a registered database, separate informed consent was not collected from the patients. Parents of the children signed and completed informed consent forms when visiting Primary Healthcare Centers (PHC), and this consent was recorded and stored in official systems. This study was also approved by the Ethics Committee of Hamadan University of Medical Sciences and has the ethics approval code IR.UMSHA.REC.1403.100. I confirm that all methos have been performed in accordance with relevant guidelines and regulations.

## Results

**Fig. 1:**
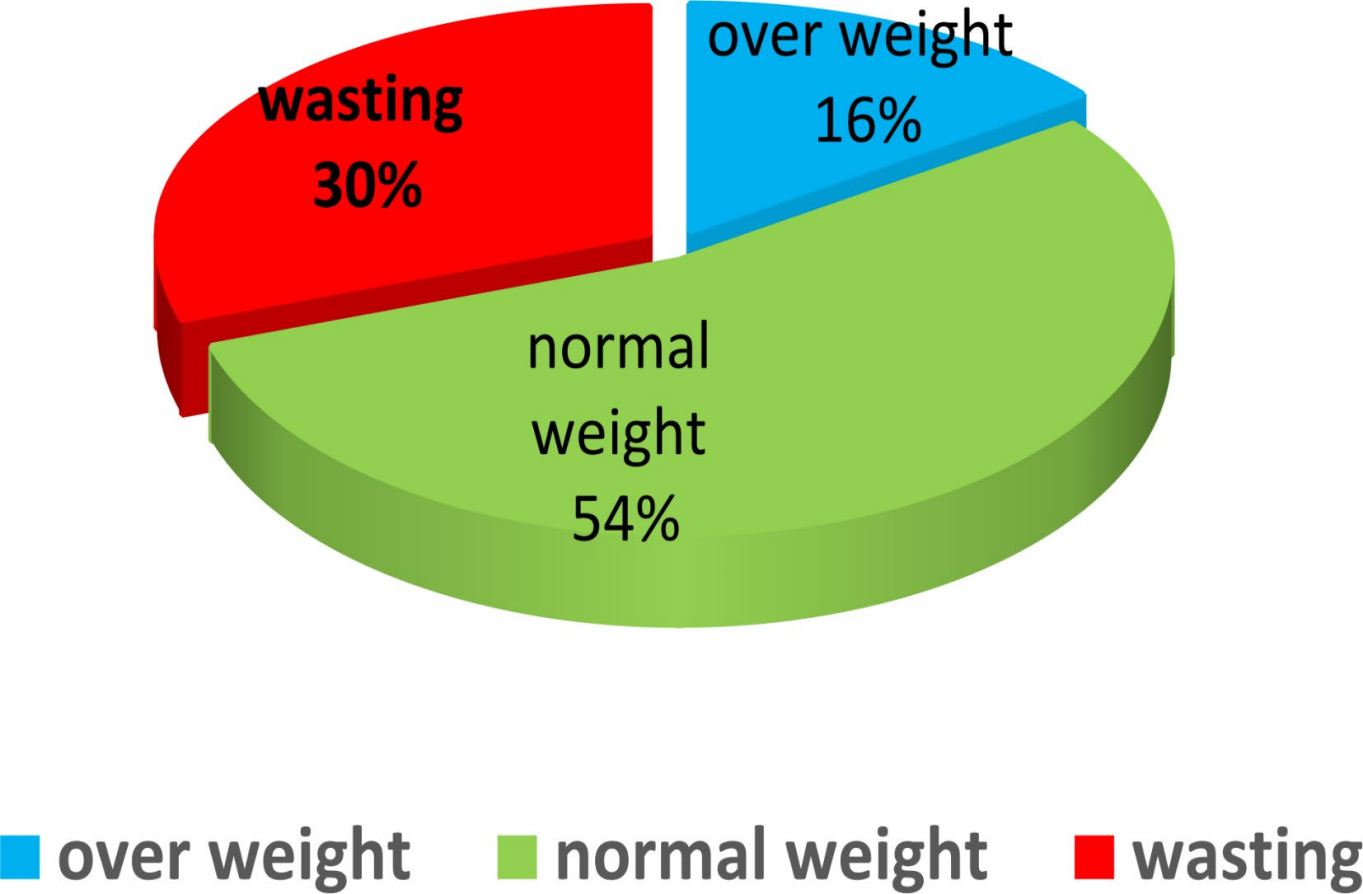
Percentage related to the response variable of weight for height.

**Fig. 2:**
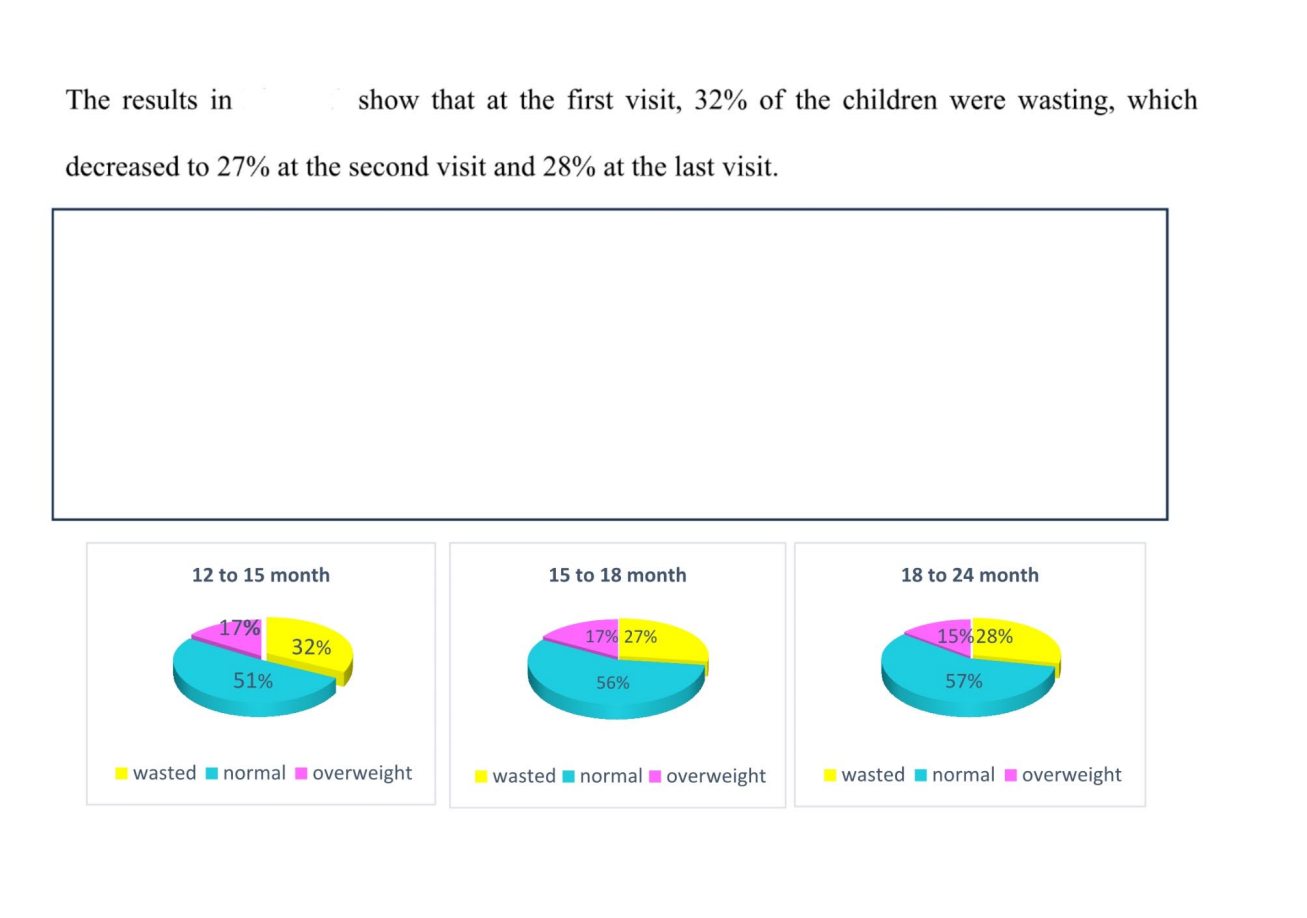
Weight gain status a cross time by response variable.

The samples examined in this longitudinal study were 455 mother-child pairs aged 12 to 24 months from the city of Hamedan, whose observations were repeated a total of 1097 times at different visits during one year (from 12 to 24 months). Figures 1 and 2 shows the percent age of the response variable weight for height of the children studied. Out of 1097 measurements, 330 observations are wasting, 590 observations are normal and 177 observations are overweight.

Table 2 presents the descriptive statistics related to 455 studied samples (mother and 1 to 2 year old child ) and also the information of 1097 references related to these 455 samples, in different months, according to different categories of weight-for-height response.

## **Socio-demographic characteristics and health status of children 1–2 years and mothers**

Table 2 shows that of the 455 children studied, 49.5% were boys and 50.5% were girls. Wasting was more common in boys (31.3%) than in girls (28.5%), with a significant gender difference (p-value = 0.025). Wasting was higher in preterm infants (55.3%) compared to term infants (25.7%), indicating a significant effect of prematurity on wasting (p-value < 0.001). Only 3.1% (14 children) were twins, and wasting was higher in twins (47.4%) compared to singletons (29.5%), indicating a significant effect of twins on wasting (p-value = 0.04). In this study, 65.7% of the children were insured. Wasting was higher in uninsured children (35.9%) compared to those with insurance (27%), indicating a significant difference (p-value = 0.005). In this study, 52.1% of the children lived the suburbs of Hamadan. Wasting was higher in suburban children (32.6%) compared to those in urban areas (27.4%), indicating a significant effect of residence on wasting (p-value < 0.001). A significant relationship was observed between the distance from the birth of the previous child and wasting(p-value = 0.013 < 0.05). Table 2 shows that the mean birth weight was significantly different among the groups: wasting children had a mean of 2851.70 ± 540.15gr, normal children 3150.59 ± 453.49gr, and overweight children 3285.93 ± 436.2 gr (p-value < 0.001).

Based on the results presented in Tables 1 and 23.8% of the mothers were under 24 years old, while 86.2% were over 24 yeas old. Wasting was more common among children of mothers under 24 years of age than among those whose mothers were older (p-value = 0.006). About 45.3% of the mothers had a university education, 30.5% had a diploma, and 24.2% had less than a diploma. About 24.8% of the mothers worked outside the home. Wasting was most common among children whose mothers had less than a diploma (p-value = 0.02). Table 2 shows that the mean age of the mothers was 33.21 ± 5.53 years, with a mean pregnancy age of 30.80 ± 5.47 years. The mean pre-pregnancy BMI was 25.70 ± 3.89 kg/m², the mean pre-pregnancy weight was 67.40 ± 10.39 kg, and the mean height was 161.60 ± 5.93 cm. Mothers of children with wasting had significantly lower mean height, weight, and pre-pregnancy BMI than mothers of children who were overweight or normal weight (p-value < 0.05).

**Table 2:** The descriptive statistics demographic characteristic of 455 mothers with children 1-2 years and 1097 references associated with these samples visits during one year

Variable	Category	N=455		N=1097		
		Frequency (%)	Wasting	Normal	Overweight	p-value
Sex child	Female	230 (50.5)	154 (28.5)	312 (57.7)	75 (13.9)	0.025
	Male	225 (49.5)	176 (31.3)	278 (50)	102 (18.3)	
Premature infants	Yes	65 (14.3)	89 (55.3)	62 (38.5)	10 (6.2)	<0.001
	No	390 (85.7)	241 (25.7)	528 (56.4)	167 (17.8)	
Nutrition style	Breastfeeding	327 (71.9)	231 (28.9)	441 (55.3)	126 (15.8)	0.26
	Formula feeding	128 (28.1)	99 (33.1)	149 (49.8)	51 (17.1)	
Birth order	1st	222 (48.4)	159 (29.1)	295 (53.9)	93 (17)	0.64
	2^nd^ and more	233 (51.6)	171 (31.1)	295 (53.6)	84 (15.3)	
Twins	Yes	14 (3.1)	18 (47.4)	17 (44.7)	3 (7.9)	0.04
	No	441 (96.9)	312(29.5)	573 (54.1)	174 (16.4)	
Insurance status of child and mother	Yes	299 (65.7)	194 (27)	397 (55.3)	127 (17.7)	0.005
	No	156 (34.3)	136 (35.9)	193 (50.9)	50 (13.2)	
Residence	Suburban	237 (52.1)	185 (32.6)	304 (53.6)	78 (13.6)	0.036
	Urban	218 (47.9)	145 (27.4)	286 (54)	99 (18.7)	
Distance from the previous child(year)	0	219 (48.1)	159 (29.5)	292 (54.2)	88 (16.3)	0.013
	01-Feb	32 (7)	22 (30.1)	41 (56.2)	10 (13.7)	
	>2	204 (44.8)	149 (30.7)	257 (53)	79 (16.3)	
Type of delivery	Natural childbirth	159 (34.9)	104 (26.9)	212 (54.9)	70 (18.1)	0.16
	Cesarean	396 (65.1)	226 (31.8)	378 (53.2)	107 (15)	
Age at pregnancy(year)	<24	63 (13.8)	58 (38.4)	80 (53)	13 (8.6)	0.006
	>24	392(86.2)	272 (28.8)	510 (53.9)	164 (17.3)	
Underlying disease mother	Yes	155 (34.1)	107 (28.4)	207 (54.9)	63 (16.7)	0.66
	No	300 (65.9)	223 (31)	383 (53.2)	114 (19.7)	
Mother’s education	<Diploma	110 (24.2)	84 (30.8)	148 (54.2)	41 (15)	0.02
	Diploma	139 (30.5)	102 (29.7)	172 (50.1)	69 (20.1)	
	Academic	206 (45.3)	144 (29.9)	270 (56.1)	67 (13.9)	
Mother occupation	Unemployed	342 (75.2)	257 (30.9)	434 (52.2)	140 (16.8)	0.18
	Employed	113 (24.8)	73 (27.4)	156 (58.6)	37 (13.9)	
History of abortion	Yes	128 (28.1)	93 (29.8)	167 (53.5)	52 (16.7)	0.95
	No	327 (71.9)	237 (30.2)	423 (53.9)	125 (15.9)	

Quantitative features	Wasting (Mean±SD)	Normal weight (Mean±SD)	Overweight (Mean±SD)	p-value
Mother’s age(year)	33.39±5.97	32.95±5.36	34.05±5.01	0.04
Pre-pregnancy weight(kg)	65.63±10.41	67.38±10.07	70.25±10.28	<0.001
mother’s height(cm)	160.53±5.46	161.75±5.64	163.37±7.55	<0.001
Pre-pregnancy BMI(kg/m^2^)	25.35±3.69	25.69±3.98	26.48±3.86	0.008
Age at prepregnancy (year)	30.92±5.89	30.56±5.28	31.5±4.91	0.12
Age child(month)	19.74±2.94	19.60±2.75	19.47±2.65	0.57
Birth weight (gr)	2851.7**±**540.15	3150.59**±**453.49	3285.9**±**436.2	<0.001

*SD* Standard Deviation

According to the results presented in Table 2, the significant variables identified by the univariate analysis are the child’s sex, prematurity, twins, insurance status of mother and child, place of residence, distance from the birth of the previous child, age at prepregnancy, mother’s education, birth weight, mother’s height, pre-pregnancy weight, pre-pregnancy BMI.

**Fig. 3:**
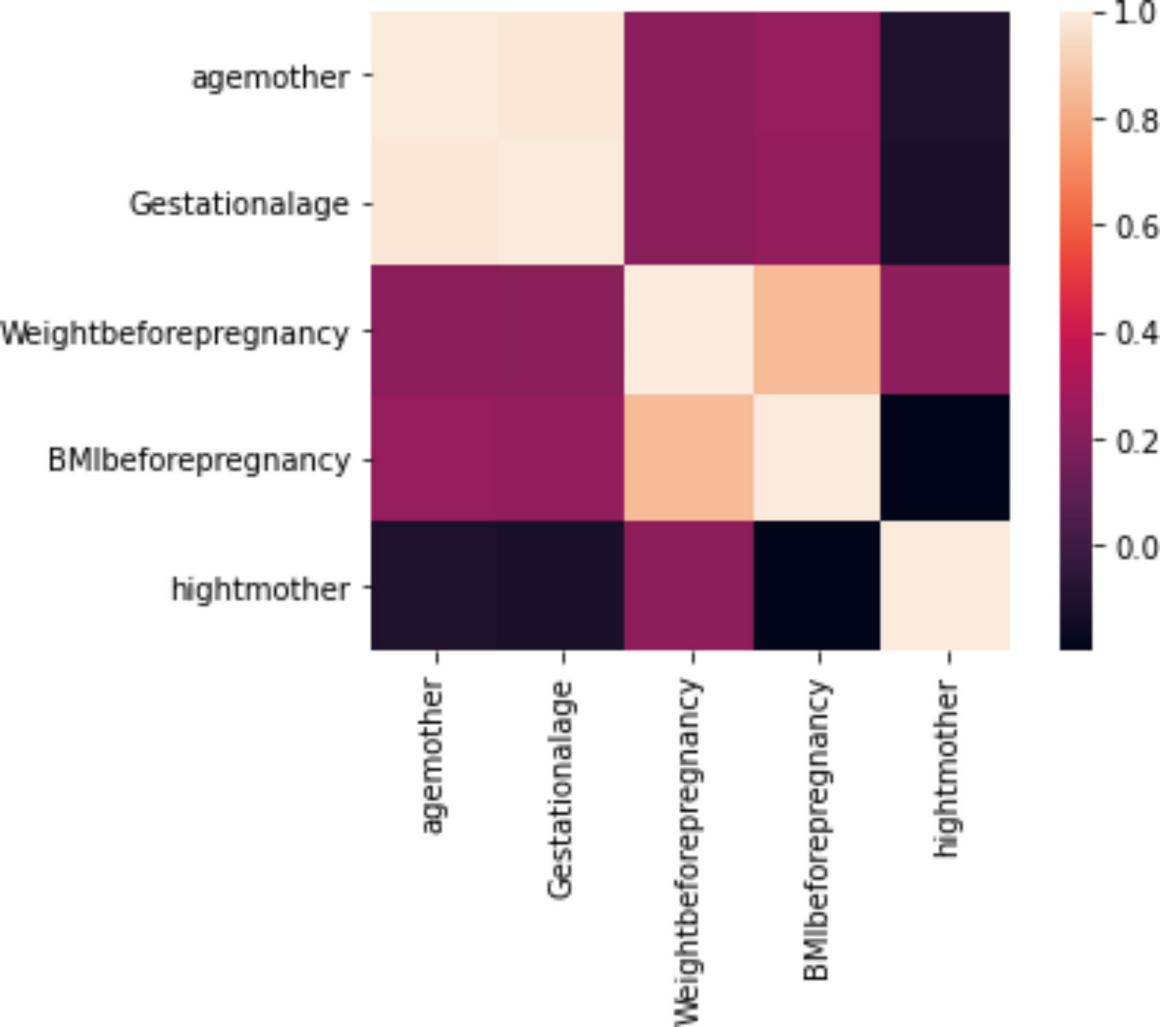
Heatmap of correlation between the quantitative variables of maternal.

To examine the correlation between the quantitative variables of age of mother, height of mother, weight before pregnancy, BMI before pregnancy, and gestational age, a heat map was used. In this heat map, each cell represents the correlation between two quantitative variables. Lighter colors indicate a higher correlation and closer to one, while darker colors indicate a lower correlation and closer to zero. According to Fig. 3, there is a high correlation between gestational age and the age mother (*r* = 0.98), and also between BMI before pregnancy and weight before pregnancy (*r* = 0.84).

After discarding the variables with high correlation, the final variables entered into the Generalized Linear Mixed Model (GLMM) included child sex, prematurity, twins, mother, and child insurance status, place of residence, distance from the birth of a previous child, mother’s gestational age, mother’s education, birth weight, mother’s height, pre-pregnancy BMI.

After identifying the significant variables based on the univariate analysis, the generalized two-level model was fitted to them, the results of which are presented in Table 3. According to the results presented in Table 3, the significant variables based on the GLMM model are the child’s birth weight, the child’s growth monitoring age, the mother’s and child’s insurance status, the mother’s and child’s place of residence, the age at prepregnancy (less than 24 years, more than 24 years) and the mother’s height.

According to the results in Table 3, the place of residence is a factor influencing wasting, so that the odds of wasting in children living in the suburban of the city are 3.80 times higher than in children living in urban areas of the city (AOR = 3.80, 95% CI: 1.69, 8.41). The insurance status in Table 3 shows that children whose mother and child did not used health insurance services were 3.09 times more likely to suffer from wasting than children who did use health insurance services (AOR = 3.09, 95% CI: 1.33–7.17). Another finding of this study was that children who were monitored at 12 to 15 months were 1.5 times more likely to lose weight than children who were monitored at 18 to 24 months (AOR = 1.15, 95% CI: 1.04–2.36). The results showed that children born to mothers younger than 24 yeas are 4.71 times more likely to be thin than children born to mothers older than 24 years (AOR = 4.71, 95% CI: 1.52,14.58). The results showed that children with higher birth weight were less likely to be wasted, such that for every 100 gr increase in a child’s birth weight, the odds of being wasted decreased by 0.77 (Adjusted Odds Ratio (AOR) = 0.77, 95% CI: 0.60–0.99). According to the results, the mother’s height has a significant effect on the child wasting. For every 1 cm increase in the mother’s height, the odds of being wasting decreased by 0.77.

**Table 3:** Unadjusted and adjusted estimates parameters model and OR (95% CI) for wasted children aged 12–24 months.

Variable	Category	Unadjusted			Adjusted		
		Estimated(SD)	OR (95% CI)	*P*-value	Estimated(SD)	OR (95% CI)	*P*-value
Premature	Ref = No	–	–	–	–	–	–
	Yes	0.71(0.18)	2.03(1.43,2.88)	0.47	0.65(0.12)	1.91(1.52,2.41)	0.56
Twin	Ref = No	–	–	–	–	–	–
	Yes	1.48(0.20)	4.39(2.97,6.48)	0.41	1.38(0.39)	4.00(1.85,8.49)	0.30
Residence	Ref = Urban	–	–	–	–	–	–
	Suburban	1.46(0.46)	4.30(1.75,10.59)	< 0.001	1.33(0.41)	3.80(1.69,8.41)	0.001
Insurance status	Ref = Yes	–	–	–	–	–	–
	No	1.10(0.40)	3(1.37,6.55)	0.003	1.13(0.43)	3.09(1.33,7.17)	0.008
Growth monitoring (month)	Ref = 18 to 24	–	–	–	–	–	–
	12 to 15	0.22(0.11)	1.24(1.01,1.53)	0.01	0.45(0.21)	1.50(1.04,2.36)	0.03
	15 to 18	0.20(0.10)	1.22(1.01,1.47)	0.17	0.40(0.27)	1.49(0.88,2.5)	0.14
Age at pregnancy	Ref > 24 year	–	–	–	–	–	–
	24year>	1.59(0.54)	4.90(1.71,14.01)	0.006	1.55(0.58)	4.71(1.52,14.58)	0.008
Birth weight(gr)	Quantitative	– 0.28(0.04)	0.75(0.70,0.81)	< 0.001	^− 0.26(0.13)^	0.77(0.60,0.99)	0.04
Mother’s height(cm)	Quantitative	– 0.04(0.05)	0.96(0.87,1.05)	< 0.001	*− 0*.08(0.02)	0.92(0.89,0.95)	0.004

*OR* Odds Ratio

*95% CI* 95% confidence interval

*SD* Standard Deviation

*Ref* Reference

## Discussion

This study aimed to investigate the risk factors associated with wasting in children of Hamadan city using a two-level ordinal regression model. Based on the results of this study, the variables of child’s birth weight, child’s growth monitoring age, mother’s and child’s insurance status, mother’s and child’s place of residence, age at pregnancy, and mother’s height were among the factors influencing the occurrence of wasting in 1–2 year old children.

Our study showed that birth weight was significantly associated with child wasting. This finding is consistent with the results of other researchers such as Aboagye in sub-Saharan Africa (AOR = 1.35)^20^, Arup Jana in India (OR = 1.33)^21^, and Asmar in The Gambia (OR = 0.98)^22^. The association between low birth weight and wasting may be due to weakened immune systems, metabolic disorders, and respiratory problems, all of which are directly related to childhood illnesses, infections, and inadequate physical growth^21^.

Another result of this research was the high rate of wasting in the suburban area of the city compared to the urban area, which was consistent with the results of the study of Akobi in Nigeria^23^ and Asim in Pakistan^24^. The reason for this problem may be the lack of facilities, and the low level of education, social, and health in the suburban compared to other urban of the city. On the other hand, Kavosi in Iran^25^ and Janevic in Serbia concluded that the prevalence of wasting in children is higher in urban areas than in rural areas. they claimed that the high cost of living in urban areas compared to rural areas has led to an increase in the poverty rate and poor nutritional status of children^26^.

Consistent with our findings, Geto in Ethiopia (AOR = 4.3)^27^, Wemakor in Ghana (AOR = 2.90)^28^ and Phuong in Bangladesh concluded that mothers who conceive at a young age are more likely to have children with wasting. Pregnancy at a young age depletes the mother’s nutrient reserves and increases the likelihood that the baby will be born malnourished. On the other hand, teenage mothers are at risk of inadequate breastfeeding and lack of experience in caring for the baby after birth, which may have an inappropriate effect on the amount and quality of child care^29^.

Based on the results of the present study, which are consistent with the studies of Porwal (OR = 1.26)^30^, Kohlmann in Nigeria^3^ and Khatun (RR = 1.28) in Bangladesh, children born to mothers with short stature are more likely to be wasted. This finding demonstrates the intergenerational link between chronic maternal and child malnutrition^31^.

Consistent with our study, Sahu in India^32^ and Baguune in Ghana concluded that regular monitoring of child growth reduces the prevalence of child wasting. Effective use of growth monitoring services enables mothers to assess their child’s health status and receive necessary advice from health workers about their children’s growth and how to improve it^33^.

Finally, according to the results of the current study, which are consistent with the studies of Aziz in Pakistan^34^, Chen in China^35^, and Nshakira in Uganda^36^, the probablity of wasting in children whose mother and child do not have health insurance is greater than that of children whose mother and child use health insurance services. The use of health insurance services reduces financial constraints related to medical expenses and increases the possibility of access to health care for both mother and child and in this sense, it has a significant positive effect on the health of the child^37^.

## Study strengths

1. One of the strengths of this study is the use of a longitudinal design, which allows changes in wasting to be tracked and observed over time.
2. A wide range of predictor variables related to both mother and child were examined, providing a comprehensive approach that allows for a more precise analysis of the factors affecting wasting and increases the validity of the findings.
3. Random selection of health centers in the city of Hamadan reduces selection bias and ensures that the study sample is representative of the general population. This randomization increases the generalizability of the study results.
4. Another strength of the study is the comparison of its results with different studies conducted in different countries. These comparisons show that our findings are consistent with international evidence, which helps to strengthen the validity of the results.

## Limitations

1. This study was conducted exclusively in the city of Hamadan, which may limit the generalizability of the results to other regions with different socio-economic and cultural backgrounds.
2. Some of the data used in the study may have been self-reported by mothers or others. Such data may be subject to personal bias or recall bias, which could affect the accuracy of the results.

## Conclusion

The results of this study suggest that most of the factors influencing child wasting can be addressed through the implementation of targeted and appropriate interventions. For example, since birth weight is one of the factors influencing wasting, early screening by health care providers can identify children at risk and facilitate timely nutritional interventions and support. Another factor influencing wasting is regular monitoring of children’s growth. Health systems need to improve access to growth monitoring services. In addition, as teenage pregnancy is associated with child wasting, the provision of adequate health care and nutritional support before, during and after pregnancy is crucial. From a policy perspective, the findings highlight the need to increase insurance coverage for low-income groups and improve access to health services in suburban areas.

## Data Availability

The dataset in this study is available at the reasonable request of the corresponding author, because the data set cannot directly access from SIB website.
